# Supplementary material for: Progressive Changes Between Thalamic Nuclei and Cortical Networks Across Stimulus–Response Learning
Source: Hum Brain Mapp. 2025 Oct 23;46(15):e70382. doi: 10.1002/hbm.70382 (PMC12547845; doi:10.1002/hbm.70382)
Supplement: Supplementary file 1 — Table S1: Detailed results for the group‐level functional connectivity analysis obtained using the Functional Network Connectivity multivariate parametric statistics implemented in the CONN toolbox. The table lists significant connectivity clusters for network pairs with p < 0.05 FDR‐corrected across clusters (in bold font) together with univariate statistics for individual connections within each cluster surviving an uncorrected threshold of p < 0.05. Table S2: Within the Focused Analysis, this table shows detailed group‐level functional connectivity analysis results obtained using a ROI‐level p‐FDR correction (ROI mass/intensity) false‐positive control method implemented in the CONN toolbox, showing mass. The table lists significant results for ROI pairs with a p‐uncorrected connection threshold of 0.05 and a cluster‐level p‐FDR corrected threshold of 0.05. [file HBM-46-e70382-s001.docx]

Supplementary Materials

**Supplementary Table 1.** Detailed results for the group-level functional connectivity analysis obtained using the Functional Network Connectivity multivariate parametric statistics implemented in the CONN toolbox. The table lists significant connectivity clusters for network pairs with p <0.05 *FDR*-corrected across clusters (in bold font) together with univariate statistics for individual connections within each cluster surviving an uncorrected threshold of p < 0.05.

| **Analysis Unit** | **Statistic** | **p-unc** | **p-FDR** |
| --- | --- | --- | --- |
| **Cluster 1/130** | **F(2,50) = 12.83** | 0.000032 | 0.004144 |
| Connection PUT-VA-L – CON | T(51) = -4.28 | 0.000082 | 0.005083 |
| Connection PUT-VA-R – CON | T(51) = -4.16 | 0.000122 | 0.009516 |
| Connection NAc-R – CON | T(51) = -3.74 | 0.000463 | 0.036119 |
| Connection NAc-shell-R – CON | T(51) = -3.72 | 0.000495 | 0.038585 |
| Connection NAc-core-L – CON | T(51) = -2.26 | 0.027800 | 0.317209 |
| **Cluster 2/130** | **F(2,50) = 11.34** | 0.000087 | 0.005658 |
| Connection FPN – CAU-body – L | T(51) = -4.93 | 0.000009 | 0.000719 |
| Connection FPN – CAU-VA – L | T(51) = -2.27 | 0.027682 | 0.093877 |
| **Cluster 3/130** | **F(2,50) = 9.56** | **0.000305** | **0.010507** |
| Connection NAc-shell-L – CAU-tail-L | T(51) = 4.51 | 0.000039 | 0.003018 |
| Connection PUT-VA-L – CAU-tail-L | T(51) = 4.14 | 0.000130 | 0.005083 |
| Connection PUT-VA-R – CAU-tail-L | T(51) = 3.44 | 0.001171 | 0.045660 |
| Connection PUT-VA-R – CAU-tail-R | T(51) = 3.12 | 0.002963 | 0.057771 |
| Connection NAc-core-R – CAU-tail-L | T(51) = 2.99 | 0.004329 | 0.104600 |
| Connection PUT-VA-L – CAU-tail-R | T(51) = 2.36 | 0.022241 | 0.108550 |
| Connection PUT-VA-R – CAU-body-R | T(51) = 2.55 | 0.013978 | 0.136289 |
| Connection NAc-shell-R – CAU-tail-L | T(51) = 2.62 | 0.011541 | 0.225059 |
| Connection NAc-core-L – CAU-tail-L | T(51) = 2.73 | 0.008744 | 0.227345 |
| **Cluster 4/130** | **F(2,50) = 9.48** | **0.000323** | **0.010507** |
| Connection Thal-L-LP – Thal-L-MDm | T(51) = 4.32 | 0.000072 | 0.005590 |
| Connection Thal-L-LP – Thal-L-VLp | T(51) = 4.02 | 0.000195 | 0.007594 |
| Connection Thal-R-LP – Thal-R-VLp | T(51) = 4.21 | 0.000103 | 0.008016 |
| Connection Thal-R-LP – Thal-L-VLp | T(51) = 3.79 | 0.000401 | 0.015649 |
| Connection Thal-L-LP – Thal-L-MDl | T(51) = 3.62 | 0.000675 | 0.017538 |
| Connection Thal-L-LP – Thal-L-VLa | T(51) = 3.44 | 0.001163 | 0.022029 |
| Connection Thal-L-LP – Thal-R-VLp | T(51) = 3.38 | 0.001412 | 0.022029 |
| Connection Thal-R-VLp – Thal-L-VLp | T(51) = 3.76 | 0.000438 | 0.034128 |
| Connection Thal-R-MDm – Thal-R-VLp | T(51) = 3.67 | 0.000577 | 0.045009 |
| Connection Thal-R-LP – Thal-L-MDl | T(51) = 3.15 | 0.002755 | 0.046021 |
| Connection Thal-R-LP – Thal-R-MDl | T(51) = 3.12 | 0.002950 | 0.046021 |
| Connection Thal-L-VLa – Thal-L-LP | T(51) = 3.66 | 0.000594 | 0.046314 |
| Connection Thal-L-LP – Thal-R-MDm | T(51) = 2.88 | 0.005775 | 0.048789 |
| Connection Thal-L-VLa – Thal-L-MDl | T(51) = 3.34 | 0.001580 | 0.061633 |
| Connection Thal-L-MDm – Thal-R-VLp | T(51) = 2.99 | 0.004245 | 0.078893 |
| Connection Thal-L-VLp – Thal-L-MDm | T(51) = 3.41 | 0.001272 | 0.099188 |
| Connection Thal-L-VLa – Thal-L-MDm | T(51) = 3.02 | 0.003888 | 0.101097 |
| Connection Thal-L-VLa – Thal-R-VLp | T(51) = 2.90 | 0.005543 | 0.108093 |
| Connection Thal-R-LP – Thal-R-MDm | T(51) = 2.43 | 0.018498 | 0.144288 |
| Connection Thal-R-LP – Thal-L-VLa | T(51) = 2.38 | 0.021070 | 0.148926 |
| Connection Thal-R-LP – Thal-L-MDm | T(51) = 2.26 | 0.027829 | 0.148926 |
| Connection Thal-L-VLa – Thal-R-LP | T(51) = 2.41 | 0.019664 | 0.153379 |
| Connection Thal-L-VLp – Thal-L-MDl | T(51) = 2.77 | 0.007745 | 0.159490 |
| Connection Thal-L-VLp – Thal-R-VLp | T(51) = 2.60 | 0.012268 | 0.159490 |
| Connection Thal-L-MDm – Thal-L-VLp | T(51) = 2.44 | 0.018416 | 0.159604 |
| Connection Thal-R-MDl – Thal-L-VLp | T(51) = 3.01 | 0.004079 | 0.196501 |
| Connection Thal-R-VLp – Thal-L-MDl | T(51) = 2.51 | 0.015327 | 0.199250 |
| Connection Thal-R-MDl – Thal-R-LP | T(51) = 2.43 | 0.018743 | 0.219273 |
| Connection Thal-R-MDm – Thal-L-VLp | T(51) = 2.57 | 0.013128 | 0.222907 |
| Connection Thal-R-MDm – Thal-R-MDl | T(51) = 2.52 | 0.014924 | 0.222907 |
| Connection Thal-R-MDm – Thal-L-MDm | T(51) = 2.32 | 0.024662 | 0.240452 |
| Connection Thal-R-VLp – Thal-R-MDl | T(51) = 2.24 | 0.029425 | 0.286895 |
| Connection Thal-R-VLp – Thal-L-VLa | T(51) = 2.18 | 0.033649 | 0.288506 |
| Connection Thal-R-VLp – Thal-L-MDm | T(51) = 2.14 | 0.036988 | 0.288506 |
| Connection Thal-L-MDm – Thal-R-LP | T(51) = 2.02 | 0.048526 | 0.290494 |
| Connection Thal-R-MDm – Thal-L-MDl | T(51) = 2.12 | 0.039050 | 0.292776 |
| Connection Thal-R-MDm – Thal-R-LP | T(51) = 2.03 | 0.047420 | 0.308228 |
| Connection Thal-L-MDl – Thal-L-MDm | T(51) = 2.05 | 0.045421 | 0.557397 |
| Connection Thal-L-MDl – Thal-L-LP | T(51) = 2.03 | 0.047075 | 0.557397 |
| **Cluster 5/130** | F(1,51) = 13.23 | 0.000642 | 0.011985 |
| Connection Language – FPN | T(51) = -3.64 | 0.000642 | 0.025037 |
| **Cluster 6/130** | F(2,50) = 8.45 | 0.000690 | 0.011985 |
| Connection SMd – AMY-l-R | T(51) = 3.23 | 0.002171 | 0.077454 |
| Connection SMd – AMY-m–L | T(51) = 3.14 | 0.002780 | 0.077454 |
| Connection SMd – AMY-m-R | T(51) = 3.08 | 0.003309 | 0.077454 |
| Connection SMd – HIP-head-m1-R | T(51) = 2.76 | 0.008053 | 0.104691 |
| Connection SMd – AMY-l-L | T(51) = 2.67 | 0.010078 | 0.112297 |
| Connection AMY-l-L – AMY-m-R | T(51) = 2.62 | 0.011491 | 0.112692 |
| Connection SMd – HIP-head-l-R | T(51) = 2.53 | 0.014443 | 0.118568 |
| Connection AMY-m-R – AMY-m-L | T(51) = 2.68 | 0.009886 | 0.128520 |
| Connection HIP-head-m1-L – AMY-m-R | T(51) = 2.35 | 0.022917 | 0.130747 |
| Connection HIP-head-m1-L – HIP-head-R | T(51) = 2.34 | 0.023467 | 0.130747 |
| Connection AMY-m-R – HIP-head-l-L | T(51) = 2.46 | 0.017424 | 0.135906 |
| Connection HIP-head-m1-L – HIP-head-l-L | T(51) = 2.28 | 0.026970 | 0.140243 |
| Connection AMY-l-L – AMY-m-L | T(51) = 2.24 | 0.029818 | 0.147354 |
| Connection HIP-head-m1-L – HIP-head-m1-R | T(51) = 2.18 | 0.034073 | 0.147400 |
| Connection HIP-head-m1-L – AMY-m-L | T(51) = 2.16 | 0.035905 | 0.147400 |
| Connection AMY-l-L – SMd | T(51) = 2.20 | 0.032272 | 0.148072 |
| Connection HIP-head-m1-R – AMY-m-R | T(51) = 2.46 | 0.017214 | 0.200160 |
| Connection HIP-head-l-L – AMY-m-R | T(51) = 2.87 | 0.005976 | 0.222108 |
| Connection HIP-head-m1-R – AMY-m-L | T(51) = 2.03 | 0.048036 | 0.288213 |
| Connection HIP-head-l-L – HIP-head-l-R | T(51) = 2.27 | 0.027186 | 0.358960 |
| Connection AMY-l-R – AMY-m-R | T(51) = 2.25 | 0.028628 | 0.366128 |
| **Cluster 7/130** | F(2,50) = 8.36 | 0.000736 | 0.011985 |
| Connection Thal-L-PuL – Thal-R-VLp | T(51) = 4.53 | 0.000036 | 0.001759 |
| Connection Thal-L-PuL – Thal-L-MDl | T(51) = 4.34 | 0.000068 | 0.001759 |
| Connection Thal-R-PuM – Thal-L-LP | T(51) = 4.56 | 0.000032 | 0.002507 |
| Connection Thal-L-PuL – Thal-L-MDm | T(51) = 3.71 | 0.000507 | 0.007914 |
| Connection Thal-L-PuL – Thal-L-VLp | T(51) = 3.60 | 0.000720 | 0.008410 |
| Connection Thal-L-PuL – Thal-R-MDl | T(51) = 3.58 | 0.000755 | 0.008410 |
| Connection Thal-L-PuL – Thal-R-MDm | T(51) = 3.40 | 0.001299 | 0.012662 |
| Connection HIP-body-R – Thal-R-MDl | T(51) = -3.84 | 0.000341 | 0.026600 |
| Connection Thal-R-CM – Thal-R-VLp | T(51) = -3.03 | 0.003884 | 0.060584 |
| Connection Thal-R-LP – Thal-L-VPL | T(51) = 2.51 | 0.015183 | 0.133682 |
| Connection Thal-L-LP – Thal-L-PuA | T(51) = 2.30 | 0.025820 | 0.143852 |
| Connection HIP-body-R – Thal-R-VLp | T(51) = -2.72 | 0.008975 | 0.144805 |
| Connection HIP-body-R – Thal-L-VLa | T(51) = -2.67 | 0.010270 | 0.144805 |
| Connection HIP-body-R – Thal-R-MDm | T(51) = -2.63 | 0.011139 | 0.144805 |
| Connection Thal-R-LP – Thal-R-PuA | T(51) = 2.24 | 0.029566 | 0.148926 |
| Connection Thal-R-LP – HIP-body-R | T(51) = -2.22 | 0.030549 | 0.148926 |
| Connection Thal-L-VLa – DAN | T(51) = -2.41 | 0.019521 | 0.153379 |
| Connection HIP-body-L – Thal-R-MDl | T(51) = -2.51 | 0.015448 | 0.156478 |
| Connection Thal-L-LP – Thal-R-PuM | T(51) = 2.20 | 0.032659 | 0.164003 |
| Connection Thal-L-LP – Thal-L-VPL | T(51) = 2.18 | 0.033642 | 0.164003 |
| Connection HIP-body-R – Thal-L-MDl | T(51) = -2.35 | 0.022706 | 0.177110 |
| Connection Thal-R-LP – PMN | T(51) = 2.11 | 0.039523 | 0.181341 |
| Connection DAN – Thal-L-VLa | T(51) = -2.79 | 0.007403 | 0.192474 |
| Connection Thal-R-MDl – HIP-body-L | T(51) = -2.87 | 0.005984 | 0.196501 |
| Connection Thal-R-MDl – HIP-body-R | T(51) = -2.67 | 0.010077 | 0.196501 |
| Connection Thal-R-LP – Thal-L-PuA | T(51) = 2.05 | 0.045649 | 0.197814 |
| Connection Thal-R-VLp – Thal-L-VPL | T(51) = 2.76 | 0.008072 | 0.199250 |
| Connection Thal-L-VLa – Thal-R-CM | T(51) = -2.24 | 0.029761 | 0.202949 |
| Connection Thal-L-LP – Thal-R-LGN | T(51) = -2.03 | 0.048114 | 0.205976 |
| Connection aGP-R – Thal-R-VLp | T(51) = 2.75 | 0.008243 | 0.207863 |
| Connection aGP-R – Thal-R-MDm | T(51) = 2.28 | 0.027108 | 0.207863 |
| Connection Thal-R-MDm – Thal-L-PuL | T(51) = 2.46 | 0.017147 | 0.222907 |
| Connection Thal-L-MDm – Thal-L-PuL | T(51) = 2.17 | 0.034817 | 0.226307 |
| Connection Thal-R-PuM – Thal-L-VLp | T(51) = 2.39 | 0.020806 | 0.226655 |
| Connection Thal-R-CM – Thal-L-MDm | T(51) = -2.29 | 0.026431 | 0.234578 |
| Connection Thal-R-MDl – Thal-L-PuA | T(51) = 2.03 | 0.047567 | 0.285400 |
| Connection Thal-R-MDm – Thal-L-PuM | T(51) = 2.09 | 0.041289 | 0.292776 |
| Connection Thal-L-CM – Thal-R-VLp | T(51) = -2.10 | 0.040517 | 0.303161 |
| Connection DAN – Thal-L-LP | T(51) = -2.15 | 0.035935 | 0.323024 |
| Connection Thal-R-PuA – Thal-L-VLp | T(51) = 2.09 | 0.041459 | 0.359308 |
| Connection Visual – Thal-L-MDm | T(51) = -2.26 | 0.028175 | 0.366275 |
| Connection Thal-L-LGN – Thal-R-MDm | T(51) = -2.38 | 0.021124 | 0.422477 |
| Connection Thal-L-LGN – Thal-R-MDl | T(51) = -2.12 | 0.038489 | 0.422477 |
| Connection Thal-R-PuL – Thal-L-VLp | T(51) = 2.45 | 0.017716 | 0.441362 |
| Connection Thal-R-PuL – Thal-L-VLp | T(51) = 2.45 | 0.017716 | 0.441362 |
| Connection Visual – Thal-R-MDm | T(51) = -2.03 | 0.047457 | 0.462709 |
| Connection Thal-L-PuM – Thal-L-LP | T(51) = 2.34 | 0.023509 | 0.549938 |
| Connection Thal-L-PuM – Thal-L-MDm | T(51) = 2.19 | 0.033370 | 0.549938 |
| Connection Thal-L-PuM – Thal-L-VLp | T(51) = 2.11 | 0.039666 | 0.549938 |
| Connection Thal-L-PuA – Thal-L-MDl | T(51) = 2.47 | 0.016818 | 0.563525 |
| **Cluster 8/130** | **F(1,51) = 12.90** | **0.000738** | **0.011985** |
| Connection SMl – CON | T(51) = -3.59 | 0.000738 | 0.019176 |
| **Cluster 9/130** | **F(2,50) = 8.07** | **0.000920** | **0.013290** |
| Connection Thal-L-PuL – CON | T(51) = 2.83 | 0.006701 | 0.040208 |
| Connection GP-p-R – CON | T(51) = 3.56 | 0.000808 | 0.063005 |
| Connection Thal-R-PuA – CON | T(51) = 3.52 | 0.000911 | 0.071078 |
| Connection CON – HIP-body-L | T(51) = -2.96 | 0.004646 | 0.120797 |
| Connection GP-a-R – CON | T(51) = 2.26 | 0.027924 | 0.207863 |
| Connection CON – Thal-R-LGN | T(51) = -2.59 | 0.012406 | 0.217671 |
| Connection CON – HIP-body-R | T(51) = -2.52 | 0.014883 | 0.217671 |
| Connection CON – Thal-L-PuA | T(51) = 2.47 | 0.016744 | 0.217671 |
| Connection CON – DAN | T(51) = 2.24 | 0.029369 | 0.254529 |
| Connection PMN – CON | T(51) = 2.36 | 0.021949 | 0.285341 |
| Connection HIP-body-L – CON | T(51) = -2.03 | 0.047801 | 0.286806 |
| Connection DAN – CON | T(51) = 2.02 | 0.048767 | 0.335462 |
| Connection Thal-L-PuI – CON | T(51) = 2.03 | 0.047452 | 0.400962 |
| Connection GP-p-L – CON | T(51) = 2.02 | 0.048859 | 0.925414 |
| **Cluster 10/130** | **F(2,50) = 7.62** | **0.001297** | **0.016661** |
| Connection DMN – Thal-R-MDm | T(51) = -3.14 | 0.002788 | 0.022539 |
| Connection DMN – Thal-R-MDl | T(51) = -2.81 | 0.007086 | 0.035002 |
| Connection DMN – Thal-L-LP | T(51) = 2.71 | 0.009191 | 0.042172 |
| Connection Thal-L-LP – DMN | T(51) = 2.59 | 0.012554 | 0.089018 |
| Connection Thal-L-MDm – DMN | T(51) = 2.45 | 0.017765 | 0.159604 |
| Connection Thal-R-MDl – DMN | T(51) = -2.18 | 0.033734 | 0.219273 |
| Connection Thal-L-MDl – DMN | T(51) = 2.32 | 0.024327 | 0.557397 |
| **Cluster 11/130** | **F(2,50) = 7.36** | **0.001574** | **0.016661** |
| Connection DMN – HIP-head-l-L | T(51) = -4.21 | 0.000103 | 0.008053 |
| Connection DMN – HIP-head-l-R | T(51) = -3.86 | 0.000320 | 0.009876 |
| Connection DMN – AMY-l-LH | T(51) = -3.44 | 0.001174 | 0.015264 |
| Connection DMN – AMY-m-L | T(51) = -3.33 | 0.001617 | 0.015766 |
| Connection DMN – AMY-l-R | T(51) = -2.80 | 0.007180 | 0.035002 |
| Connection DMN – HIP-head-m1-L | T(51) = -2.59 | 0.012628 | 0.052874 |
| Connection DMN – AMY-m-R | T(51) = -2.49 | 0.016084 | 0.059740 |
| **Cluster 12/130** | **F(2,50) = 7.18** | **0.001813** | **0.016661** |
| Connection HIP-tail-R – HIP-head-m2-R | T(51) = 4.21 | 0.000103 | 0.004021 |
| Connection HIP-tail-L – HIP-head-m2-R | T(51) = 3.74 | 0.000474 | 0.020284 |
| Connection Thal-L-PuI – HIP-head-m2-R | T(51) = 3.14 | 0.002797 | 0.109092 |
| Connection HIP-head-m2-L – HIP-tail-L | T(51) = 3.33 | 0.001612 | 0.109826 |
| Connection HIP-head-m2-L – PMN | T(51) = -3.14 | 0.002816 | 0.109826 |
| Connection HIP-head-m2-L – HIP-tail-R | T(51) = 2.95 | 0.004815 | 0.125195 |
| Connection HIP-body-L – HIP-head-m2-R | T(51) = 2.49 | 0.016049 | 0.156478 |
| Connection HIP-head-m2-R – HIP-body-L | T(51) = 3.15 | 0.002717 | 0.201472 |
| Connection HIP-head-m2-R – HIP-tail-R | T(51) = 2.91 | 0.005334 | 0.201472 |
| Connection HIP-head-m2-R – HIP-body-R | T(51) = 2.77 | 0.007749 | 0.201472 |
| Connection Thal-L-CM – HIP-head-m2-L | T(51) = 2.31 | 0.024764 | 0.241448 |
| Connection HIP-body-R – HIP-head-m2-L | T(51) = 2.12 | 0.038764 | 0.251345 |
| Connection HIP-head-m2-L – Thal-R-CM | T(51) = 2.35 | 0.022936 | 0.277534 |
| Connection HIP-body-L – HIP-head-m2-L | T(51) = 2.06 | 0.044523 | 0.286806 |
| Connection HIP-head-m2-L – HIP-body-L | T(51) = 2.16 | 0.035407 | 0.298794 |
| Connection Thal-L-CM – HIP-head-m2-R | T(51) = 2.01 | 0.049791 | 0.303161 |
| Connection HIP-head-m2-L – HIP-body-R | T(51) = 2.04 | 0.046661 | 0.303297 |
| Connection HIP-head-m2-R – Thal-R-PuM | T(51) = -2.48 | 0.016647 | 0.324610 |
| Connection HIP-head-m2-R – HIP-tail-L | T(51) = 2.34 | 0.023204 | 0.361986 |
| Connection HIP-head-m2-R – Thal-L-PuM | T(51) = -2.12 | 0.038521 | 0.382523 |
| Connection HIP-head-m2-R – Thal-R-PuL | T(51) = -2.12 | 0.039233 | 0.382523 |
| **Cluster 13/130** | **F(2,50) = 7.17** | **0.001822** | **0.016661** |
| Connection CAU-tail-L – CON | T(51) = 3.56 | 0.000825 | 0.010722 |
| Connection CAU-body-R – CON | T(51) = 3.13 | 0.002887 | 0.028144 |
| Connection CAU-tail-R – CON | T(51) = 2.73 | 0.008749 | 0.341225 |
| **Cluster 14/130** | **F(2,50) = 7.12** | **0.001909** | **0.016661** |
| Connection Thal-R-CM – AMY-m-R | T(51) = 3.99 | 0.000214 | 0.016682 |
| Connection HIP-head-m1-L – HIP-body-L | T(51) = 3.54 | 0.000854 | 0.025362 |
| Connection HIP-head-m1-L – HIP-body-R | T(51) = 3.46 | 0.001090 | 0.025362 |
| Connection HIP-head-m1-L – GP-p-L | T(51) = 3.16 | 0.002657 | 0.036745 |
| Connection HIP-body-L – AMY-m-R | T(51) = 3.16 | 0.002651 | 0.058085 |
| Connection Thal-R-CM – AMY-l-R | T(51) = 3.36 | 0.001497 | 0.058370 |
| Connection Thal-R-CM – AMY-l-L | T(51) = 3.13 | 0.002896 | 0.060584 |
| Connection HIP-body-L – AMY-l-L | T(51) = 2.99 | 0.004318 | 0.067354 |
| Connection AMY-m-R – HIP-body-R | T(51) = 3.35 | 0.001516 | 0.067605 |
| Connection AMY-m-R – Thal-L-VPL | T(51) = -3.31 | 0.001733 | 0.067605 |
| Connection PMN – HIP-head-l-R | T(51) = -3.54 | 0.000877 | 0.068392 |
| Connection DAN – AMY-l-R | T(51) = 3.52 | 0.000924 | 0.072100 |
| Connection PMN – HIP-head-l-L | T(51) = -3.21 | 0.002270 | 0.088548 |
| Connection HIP-head-l-L – HIP-body-L | T(51) = 3.45 | 0.001143 | 0.089116 |
| Connection AMY-m-R – HIP-body-L | T(51) = 2.99 | 0.004270 | 0.091868 |
| Connection AMY-m-R – PMN | T(51) = -2.90 | 0.005444 | 0.091868 |
| Connection HIP-head-m1-L – GP-p-R | T(51) = 2.65 | 0.010580 | 0.096922 |
| Connection HIP-head-m1-L – Thal-L-CM | T(51) = 2.59 | 0.012426 | 0.096922 |
| Connection Thal-R-CM – AMY-m-L | T(51) = 2.74 | 0.008491 | 0.110378 |
| Connection AMY-l-L – GP-a-L | T(51) = -2.72 | 0.008970 | 0.112692 |
| Connection AMY-l-L – DAN | T(51) = 2.49 | 0.015892 | 0.112692 |
| Connection AMY-l-L – Thal-L-PuM | T(51) = -2.37 | 0.021798 | 0.130787 |
| Connection PMN – AMY-m-R | T(51) = -2.88 | 0.005739 | 0.135909 |
| Connection PMN – HIP-head-m1-L | T(51) = -2.73 | 0.008712 | 0.135909 |
| Connection Visual – HIP-head-m1-R | T(51) = 2.80 | 0.007194 | 0.140287 |
| Connection HIP-body-R – AMY-m-L | T(51) = 2.88 | 0.005857 | 0.144805 |
| Connection HIP-body-R – AMY-m-R | T(51) = 2.86 | 0.006179 | 0.144805 |
| Connection HIP-body-L – AMY-m-L | T(51) = 2.58 | 0.012667 | 0.156478 |
| Connection HIP-body-L – Thal-L-PuM | T(51) = -2.11 | 0.040189 | 0.156738 |
| Connection HIP-head-L – HIP-tail-L | T(51) = 2.07 | 0.043780 | 0.162610 |
| Connection AMY-l-L – Thal-R-CM | T(51) = 2.06 | 0.045017 | 0.167312 |
| Connection HIP-body-R – HIP-head-l-R | T(51) = 2.39 | 0.020334 | 0.176231 |
| Connection HIP-head-m1-R – HIP-tail-R | T(51) = 2.60 | 0.012151 | 0.200160 |
| Connection HIP-head-m1-R – HIP-body-R | T(51) = 2.56 | 0.013335 | 0.200160 |
| Connection HIP-head-m1-R – PON | T(51) = 2.54 | 0.014233 | 0.200160 |
| Connection AMY-m-R – Thal-R-PuA | T(51) = -2.12 | 0.039308 | 0.216094 |
| Connection Thal-L-CM – AMY-m-L | T(51) = 2.60 | 0.012135 | 0.216833 |
| Connection Thal-L-CM – HIP-head-m1-R | T(51) = 2.50 | 0.015734 | 0.216833 |
| Connection Thal-L-CM-SS – AMY-m-R | T(51) = 2.48 | 0.016679 | 0.216833 |
| Connection HIP-head-m1-R – HIP-body-L | T(51) = 2.35 | 0.022431 | 0.218705 |
| Connection HIP-head-l-L – Thal-R-CM | T(51) = 2.74 | 0.008543 | 0.222108 |
| Connection Thal-L-CM – HIP-head-m1-L | T(51) = 2.33 | 0.023902 | 0.241448 |
| Connection HIP-body-R – HIP-head-m1-R | T(51) = 2.11 | 0.039439 | 0.251345 |
| Connection Thal-R-PuL – HIP-head-l-L | T(51) = -3.08 | 0.003286 | 0.256325 |
| Connection Thal-R-LGN – AMY-l-R | T(51) = 2.22 | 0.030629 | 0.269010 |
| Connection Thal-R-LGN – AMY-l-L | T(51) = 2.10 | 0.041030 | 0.269010 |
| Connection HIP-tail-R – AMY-m-R | T(51) = 2.05 | 0.045974 | 0.275842 |
| Connection Thal-L-CM – AMY-l-R | T(51) = 2.08 | 0.042841 | 0.303161 |
| Connection DAN – HIP-head-m1-R | T(51) = 2.37 | 0.021360 | 0.323024 |
| Connection DAN – AMY-m-L | T(51) = 2.28 | 0.026818 | 0.323024 |
| Connection GP-p-R – AMY-l-L | T(51) = 2.37 | 0.021392 | 0.353543 |
| Connection HIP-head-l-L – HIP-body-R | T(51) = 2.20 | 0.032214 | 0.358960 |
| Connection AMY-l-R – Thal-L-PuM | T(51) = -2.61 | 0.011970 | 0.366128 |
| Connection AMY-l-R – DAN | T(51) = 2.23 | 0.030416 | 0.366128 |
| Connection AMY-l-R – GP-p-L | T(51) = 2.09 | 0.041650 | 0.366128 |
| Connection AMY-l-R – Thal-R-VPL | T(51) = -2.03 | 0.047332 | 0.366128 |
| Connection PON – HIP-head-l-R | T(51) = 2.16 | 0.035653 | 0.373809 |
| Connection PON – AMY-m-L | T(51) = 2.13 | 0.038169 | 0.373809 |
| Connection PON – AMY-m-R | T(51) = 2.07 | 0.043132 | 0.373809 |
| Connection Visual – AMY-l-R | T(51) = 2.18 | 0.033609 | 0.374497 |
| Connection HIP-head-l-L – PMN | T(51) = -2.06 | 0.044349 | 0.378178 |
| Connection Thal-L-LGN – AMY-l-R | T(51) = 2.69 | 0.009759 | 0.422477 |
| Connection Thal-R-PuL – HIP-head-m1-L | T(51) = -2.15 | 0.036318 | 0.441362 |
| Connection HIP-tail-L – HIP-head-l-R | T(51) = 2.07 | 0.043409 | 0.457540 |
| Connection AMY-m-L – PMN | T(51) = -2.39 | 0.020725 | 0.497065 |
| Connection AMY-m-L – DAN | T(51) = 2.36 | 0.021909 | 0.497065 |
| Connection AMY-m-L – GP-a-L | T(51) = -2.13 | 0.038026 | 0.497065 |
| Connection AMY-m-L – Thal-L-VPL | T(51) = -2.06 | 0.044390 | 0.497065 |
| Connection HIP-head-l-R – DAN | T(51) = 2.74 | 0.008484 | 0.499825 |
| Connection HIP-head-l-R – PON | T(51) = 2.40 | 0.019930 | 0.499825 |
| **Cluster 15/130** | **F(2,50) = 7.11** | **0.001922** | **0.016661** |
| Connection DMN – HIP-body-R | T(51) = -3.81 | 0.000380 | 0.009876 |
| Connection DMN – HIP-tail-L | T(51) = -3.60 | 0.000712 | 0.013883 |
| Connection DMN – Thal-R-Pul | T(51) = -3.45 | 0.001127 | 0.015264 |
| Connection DMN – Thal-R-PuM | T(51) = -3.36 | 0.001482 | 0.015766 |
| Connection DMN – Visual | T(51) = -3.13 | 0.002890 | 0.022539 |
| Connection DMN – HIP-tail-R | T(51) = -2.95 | 0.004762 | 0.031368 |
| Connection DMN – HIP-body-L | T(51) = -2.93 | 0.005070 | 0.031368 |
| Connection DMN – PMN | T(51) = -2.85 | 0.006222 | 0.034663 |
| Connection DMN – Thal-R-CM-SS-8206 | T(51) = -2.58 | 0.012880 | 0.052874 |
| Connection PMN – DMN | T(51) = -2.78 | 0.007555 | 0.135909 |
| Connection Thal-R-CM – DMN | T(51) = -2.29 | 0.026098 | 0.234578 |
| Connection DAN – DMN | T(51) = -2.11 | 0.040124 | 0.323024 |
| Connection Thal-R-PuA – DMN | T(51) = -2.24 | 0.029819 | 0.344350 |
| Connection Thal-R-PuL – DMN | T(51) = -2.04 | 0.046561 | 0.441362 |
| Connection Thal-L-PuM – DMN | T(51) = 2.43 | 0.018569 | 0.549938 |
| **Cluster 16/130** | **F(2,50) = 6.44** | **0.003248** | **0.026394** |
| Connection FPN – Thal-L-VA | T(51) = -2.94 | 0.004893 | 0.051191 |
| Connection FPN – PUT-VP-R | T(51) = 2.88 | 0.005825 | 0.051191 |
| Connection FPN – Thal-R-VA | T(51) = -2.85 | 0.006280 | 0.051191 |
| Connection PUT-VP-R – FPN | T(51) = 2.86 | 0.006112 | 0.059595 |
| Connection FPN – Thal-R-VLa | T(51) = -2.68 | 0.009880 | 0.060242 |
| Connection FPN – PUT-DP-R | T(51) = 2.52 | 0.014874 | 0.077343 |
| **Cluster 17/130** | **F(2,50) = 5.90** | **0.004991** | **0.037757** |
| Connection FPN – Thal-R-MDl | T(51) = -4.23 | 0.000099 | 0.003852 |
| Connection FPN – Thal-R-VLp | T(51) = -3.50 | 0.000982 | 0.019143 |
| Connection FPN – Thal-R-MDm | T(51) = -3.15 | 0.002716 | 0.037592 |
| Connection FPN – Thal-L-MDl | T(51) = -2.45 | 0.017674 | 0.081577 |
| Connection FPN – Thal-L-MDm | T(51) = -2.38 | 0.020858 | 0.081577 |
| Connection FPN – Thal-L-VLa | T(51) = -2.28 | 0.027079 | 0.093877 |
| Connection Thal-L-LP – FPN | T(51) = -2.46 | 0.017529 | 0.105174 |
| **Cluster 18/130** | **F(1,51) = 8.52** | **0.005228** | **0.037757** |
| Connection DMN – CON | T(51) = -2.92 | 0.005228 | 0.031368 |
| Connection CON – DMN | T(51) = -3.05 | 0.003640 | 0.120797 |
| **Cluster 19/130** | **F(2,50) = 5.65** | **0.006120** | **0.041875** |
| Connection Thal-L-CeM – SAN | T(51) = 2.23 | 0.030425 | 0.469049 |
| **Cluster 20/130** | **F(2,50) = 5.40** | **0.007535** | **0.047821** |
| Connection CAU-body-L – CAU-tail-R | T(51) = 2.69 | 0.009531 | 0.082606 |
| Connection CAU-body-L – CAU-body-R | T(51) = 2.59 | 0.012505 | 0.097538 |
| Connection CAU-VA-L – CAU-tail-L | T(51) = 2.86 | 0.006046 | 0.127264 |
| Connection CAU-VA-L – CAU-tail-R | T(51) = 2.72 | 0.008953 | 0.127264 |
| Connection CAU-VA-R – CAU-tail-L | T(51) = 2.65 | 0.010713 | 0.233022 |
| Connection CAU-VA-R – CAU-tail-R | T(51) = 2.52 | 0.014937 | 0.233022 |
| **Cluster 21/130** | **F(2,50) = 5.37** | **0.007725** | **0.047821** |
| Connection CAU-VA-L – SAN | T(51) = 4.09 | 0.000152 | 0.011822 |
| Connection CAU-body-L – SAN | T(51) = 2.39 | 0.020504 | 0.145395 |
| Connection CAU-VA-R – SAN | T(51) = 2.09 | 0.041257 | 0.321075 |
| **Cluster 22/130** | **F(2,50) = 5.28** | **0.008340** | **0.049284** |
| Connection FPN – CAU-body-R | T(51) = -3.68 | 0.000564 | 0.014662 |
| Connection FPN – CAU-tail-L | T(51) = -2.32 | 0.024528 | 0.091103 |
| Connection CAU-tail-L – FPN | T(51) = -2.35 | 0.022704 | 0.160994 |

**Supplementary Table 2.** Within the Focused Analysis, this table shows detailed group-level functional connectivity analysis results obtained using a ROI-level p-FDR correction (ROI mass/intensity) false-positive control method implemented in the CONN toolbox, showing mass. The table lists significant results for ROI pairs with a *p*-uncorrected connection threshold of 0.05 and a cluster-level p-FDR corrected threshold of 0.05.

| **Analysis Unit** | **Statistic** | **p-unc** | **p-FDR** |
| --- | --- | --- | --- |
| **Cluster 1/38 – Thal-L-PuL**  Mass = 169.69, 0.000039, 0.001488, 0.001000 |  |  |  |
| Connection Thal-L-PuL – Thal-R-VLp | T(51) = 4.53 | 0.000036 | 0.020154 |
| Connection Thal-L-PuL – Thal-R-PuA | T(51) = 4.36 | 0.000063 | 0.020154 |
| Connection Thal-L-PuL – Thal-L-MDl | T(51) = 4.34 | 0.000068 | 0.020154 |
| Connection Thal-L-PuL – Thal-R-PuM | T(51) = 3.72 | 0.000500 | 0.042250 |
| Connection Thal-L-PuL – Thal-L-MDm | T(51) = 3.71 | 0.000507 | 0.042250 |
| Connection Thal-L-PuL – Thal-L-VLp | T(51) = 3.60 | 0.000720 | 0.044006 |
| Connection Thal-L-PuL – Thal-R-MDl | T(51) = 3.58 | 0.000755 | 0.044218 |
| Connection Thal-L-PuL – Thal-R-MDm | T(51) = 3.40 | 0.001299 | 0.065210 |
| Connection Thal-L-PuL – Thal-L-PuM | T(51) = 3.23 | 0.002167 | 0.087049 |
| Connection Thal-L-PuL – Thal-R-VPL | T(51) = 3.19 | 0.002448 | 0.095591 |
| Connection Thal-L-PuL – Thal-R-PuL | T(51) = 2.92 | 0.005240 | 0.117656 |
| Connection Thal-L-PuL – Thal-L-PuA | T(51) = 2.66 | 0.010539 | 0.176399 |
| Connection Thal-L-PuL – Thal-R-VLa | T(51) = 2.31 | 0.024770 | 0.263833 |
| Connection Thal-L-PuL – Thal-L-VPL | T(51) = 2.18 | 0.033589 | 0.301342 |
| **ROI 2/38 – Thal-L-LP**  Mass = 122.20, 0.000260, 0.004946, 0.009000 |  |  |  |
| Connection Thal-L-LP – Thal-L-MDm | T(51) = 4.32 | 0.000072 | 0.020154 |
| Connection Thal-L-LP – Thal-L-VLp | T(51) = 4.02 | 0.000195 | 0.030420 |
| Connection Thal-L-LP – Thal-L-MDl | T(51) = 3.62 | 0.000675 | 0.044006 |
| Connection Thal-L-LP – Thal-L-VLa | T(51) = 3.44 | 0.001163 | 0.062884 |
| Connection Thal-L-LP – Thal-R-VLp | T(51) = 3.38 | 0.001412 | 0.068463 |
| Connection Thal-L-LP – Thal-L-CeM | T(51) = 3.01 | 0.004055 | 0.106215 |
| Connection Thal-L-LP – Thal-L-AV | T(51) = 2.88 | 0.005764 | 0.119417 |
| Connection Thal-L-LP – Thal-R-MDm | T(51) = 2.88 | 0.005775 | 0.119417 |
| Connection Thal-L-LP – Thal-L-VA | T(51) = 2.53 | 0.014518 | 0.210431 |
| Connection Thal-L-LP – Thal-L-PuA | T(51) = 2.30 | 0.025820 | 0.269917 |
| Connection Thal-L-LP – Thal-R-PuM | T(51) = 2.20 | 0.032659 | 0.301342 |
| Connection Thal-L-LP – Thal-L-VPL | T(51) = 2.18 | 0.033642 | 0.301342 |
| Connection Thal-L-LP – Thal-R-LGN | T(51) = -2.03 | 0.048114 | 0.330778 |
| **ROI 3/38 – PUT-VA**  Mass = 107.97, 0.000658, 0.008333, 0.022000 |  |  |  |
| Connection PUT-VA-LH – Thal-R-VLp | T(51) = 3.63 | 0.000649 | 0.044006 |
| Connection PUT-VA-LH – PUT-DA-LH | T(51) = 3.61 | 0.000689 | 0.044006 |
| Connection PUT-VA-LH – Thal-L-MDm | T(51) = 3.09 | 0.003217 | 0.100906 |
| Connection PUT-VA-LH – PUT-VP-RH | T(51) = 2.99 | 0.004301 | 0.106798 |
| Connection PUT-VA-LH – PUT-DA-RH | T(51) = 2.98 | 0.004406 | 0.106798 |
| Connection PUT-VA-LH – Thal-R-LP | T(51) = 2.90 | 0.005456 | 0.119417 |
| Connection PUT-VA-LH – Thal-R-MDl | T(51) = 2.80 | 0.007144 | 0.140061 |
| Connection PUT-VA-LH – Thal-L-VLp | T(51) = 2.49 | 0.016260 | 0.215442 |
| Connection PUT-VA-LH – PUT-VP-LH | T(51) = 2.40 | 0.020284 | 0.241694 |
| Connection PUT-VA-LH – PUT-DP-RH | T(51) = 2.34 | 0.023221 | 0.259873 |
| Connection PUT-VA-LH – PUT-DP-LH | T(51) = 2.33 | 0.023658 | 0.259873 |
| Connection PUT-VA-LH – Thal-L-AV | T(51) = 2.28 | 0.026871 | 0.273770 |
| Connection PUT-VA-LH – Thal-L-MDl | T(51) = 2.26 | 0.028139 | 0.280593 |
| Connection PUT-VA-LH – Thal-R-MDm | T(51) = 2.22 | 0.031194 | 0.292396 |
| **ROI 4/38 – Thal-R-LP**  Mass = 93.84, 0.001400, 0.013300, 0.045000 |  |  |  |
| Connection Thal-R-LP – Thal-R-VLp | T(51) = 4.21 | 0.000103 | 0.024081 |
| Connection Thal-R-LP – Thal-L-VLp | T(51) = 3.79 | 0.000401 | 0.042250 |
| Connection Thal-R-LP – Thal-L-MDl | T(51) = 3.15 | 0.002755 | 0.096835 |
| Connection Thal-R-LP – Thal-R-VLa | T(51) = 3.14 | 0.002826 | 0.096918 |
| Connection Thal-R-LP – Thal-R-MDl | T(51) = 3.12 | 0.002950 | 0.098756 |
| Connection Thal-R-LP – Thal-L-VPL | T(51) = 2.51 | 0.015183 | 0.211271 |
| Connection Thal-R-LP – Thal-R-MDm | T(51) = 2.43 | 0.018498 | 0.226163 |
| Connection Thal-R-LP – Thal-L-VLa | T(51) = 2.38 | 0.021070 | 0.243446 |
| Connection Thal-R-LP – Thal-L-MDm | T(51) = 2.26 | 0.027829 | 0.279479 |
| Connection Thal-R-LP – Thal-R-PuA | T(51) = 2.24 | 0.029566 | 0.284654 |
| Connection Thal-R-LP – Thal-L-PuA | T(51) = 2.05 | 0.045649 | 0.330778 |
| **ROI 5/38 – Thal-R-PuM**  Mass = 80.41, 0.003026, 0.023000, 0.088000 |  |  |  |
| Connection Thal-R-PuM – Thal-L-LP | T(51) = 4.56 | 0.000032 | 0.020154 |
| Connection Thal-R-PuM – Thal-L-PuL | T(51) = 3.92 | 0.000267 | 0.034152 |
| Connection Thal-R-PuM – Thal-L-VPL | T(51) = 3.17 | 0.002543 | 0.096636 |
| Connection Thal-R-PuM – Thal-R-VA | T(51) = -2.76 | 0.007970 | 0.151325 |
| Connection Thal-R-PuM – Thal-R-LGN | T(51) = 2.75 | 0.008226 | 0.152189 |
| Connection Thal-R-PuM – Thal-L-VLp | T(51) = 2.39 | 0.020806 | 0.243446 |
| Connection Thal-R-PuM – Thal-R-PuL | T(51) = 2.18 | 0.034028 | 0.302807 |
| Connection Thal-R-PuM – Thal-L-PuA | T(51) = 2.09 | 0.041646 | 0.321723 |
| Connection Thal-R-PuM – Thal-L-VA | T(51) = -2.05 | 0.045947 | 0.330778 |
| **ROI 6/38 – PUT-DP-RH**  Mass = 76.38, 0.003822, 0.024208, 0.113000 |  |  |  |
| Connection PUT-DP-RH – PUT-DA-LH | T(51) = 4.11 | 0.000145 | 0.025796 |
| Connection PUT-DP-RH – PUT-DA-RH | T(51) = 4.10 | 0.000147 | 0.025796 |
| Connection PUT-DP-RH – PUT-VP-LH | T(51) = 3.49 | 0.001010 | 0.056817 |
| Connection PUT-DP-RH – Thal-R-PuA | T(51) = -2.73 | 0.008709 | 0.156979 |
| Connection PUT-DP-RH – Thal-L-AV | T(51) = 2.68 | 0.009853 | 0.168938 |
| Connection PUT-DP-RH – PUT-VP-RH | T(51) = 2.54 | 0.014031 | 0.205503 |
| Connection PUT-DP-RH – Thal-R-CM | T(51) = -2.29 | 0.026229 | 0.271158 |
| Connection PUT-DP-RH – Thal-R-AV | T(51) = 2.04 | 0.046797 | 0.330778 |
| **ROI 7/38 – Thal-R-MDm**  Mass = 71.47, 0.005289, 0.028714, 0.153000 |  |  |  |
| Connection Thal-R-MDm – Thal-R-VLp | T(51) = 3.67 | 0.000577 | 0.043939 |
| Connection Thal-R-MDm – Thal-R-AV | T(51) = 3.15 | 0.002722 | 0.096835 |
| Connection Thal-R-MDm – Thal-L-VLp | T(51) = 2.57 | 0.013128 | 0.200630 |
| Connection Thal-R-MDm – Thal-R-MDl | T(51) = 2.52 | 0.014924 | 0.211271 |
| Connection Thal-R-MDm – Thal-L-PuL | T(51) = 2.46 | 0.017147 | 0.221177 |
| Connection Thal-R-MDm – Thal-R-VLa | T(51) = 2.37 | 0.021854 | 0.249815 |
| Connection Thal-R-MDm – Thal-L-MDm | T(51) = 2.32 | 0.024662 | 0.263833 |
| Connection Thal-R-MDm – Thal-L-AV | T(51) = 2.25 | 0.028577 | 0.282956 |
| Connection Thal-R-MDm – Thal-L-MDl | T(51) = 2.12 | 0.039050 | 0.321723 |
| Connection Thal-R-MDm – Thal-L-PuM | T(51) = 2.09 | 0.041289 | 0.321723 |
| Connection Thal-R-MDm – Thal-R-LP | T(51) = 2.03 | 0.047420 | 0.330778 |
